# Comparative Study of the Adverse Events Associated With Adjuvant Use of Dexmedetomidine and Clonidine in Local Anesthesia

**DOI:** 10.3389/fmed.2021.602966

**Published:** 2021-06-24

**Authors:** Jinjin Jiang, Huasu Shen, Jin Zhang, Zhen Wu, Xian Shao, Jingjing Cui, Bao Zhang, Xiaoyu Ma

**Affiliations:** ^1^Department of Anesthesiology, Shaoxing Shangyu Maternal and Child Health Hospital, Shaoxing, China; ^2^Department of Anesthesiology, The Fourth Hospital of Shijiazhuang, Shijiazhuang, China; ^3^Department of Anesthesiology, The Fourth Hospital of Hebei Medical University, Shijiazhuang, China; ^4^Department of Anesthesiology, Cangzhou Hospital of Integrated TCM-WM Hebei, Cangzhou, China

**Keywords:** dexmedetomidine, clonidine, anesthesia, regional, local, alpha-2 agonists

## Abstract

**Background:** Although clonidine and dexmedetomidine are used as alpha-2 agonists to improve the quality and duration of blockade induced by local anesthetics, no study has been reported to compare their associated adverse events in local anesthesia. The aim of this study is to compare the adverse events associated with the adjuvant use of dexmedetomidine and clonidine in local anesthesia.

**Methods:** A comprehensive search was performed to retrieve any reported adverse event associated with adjuvant use of dexmedetomidine and clonidine in local anesthesia from published literature up to 1 July 2020. Assessment of the quality of included studies was performed by the Jadad score. A comparison of any reported adverse event was made between interventions by pooling data from studies using a direct meta-analysis technique. Dichotomous outcomes were summarized as risk ratios. The review was performed according to PRISMA guideline.

**Results:** From 121 articles retrieved from the search finally 14 articles including 1,120 patients had eligibility criteria for including in the meta-analysis. No significant difference was observed between bradycardia/hypotension (OR = 1.17; 95 % CI = 0.66–2.10; *P* = 0.580; *I*^2^ = 53.78 %, *P* = 0.027), nausea/vomiting (OR = 0.91; 95% CI = 0.59-1.42; *P* = 0.706; *I*^2^ = 0.0 %, *P* = 0.940) dizziness/headache (OR = 1.10; 95% CI = 0.44–2.75; *P* = 0.831; *I*^2^ = 0.0 %, *P* = 0.882) shivering (OR = 0.95 % CI = 0.50–1.66; *P* = 0.831; *I*^2^ = 0.0 %, *P* = 0.920) and dry mouth (OR = 1.00; 95 % CI = 0.50–1.96; *P* = 0.996; *I*^2^ = 0.0%, *P* = 0.900). No significant difference was observed in subgroup comparison of adverse events in the intravenous or local adjuvant use of the study drugs (*p* > 0.05).

**Conclusion:** There is no difference in adverse events associated with the intravenous or local adjuvant use of dexmedetomidine and clonidine in local anesthesia.

## Introduction

Proper pain management with minimal side effects in patients under local anesthesia showed to improve the outcomes of local anesthesia. Studies showed that this goal is partly achieved by improving the performance of local anesthetics with adjuvant drugs such as narcotics and alpha-2 adrenoreceptor agonists to induce longer postoperative analgesia and to minimize intraoperative blood loss and postoperative side effects. Adjuvants such as morphine, fentanyl, clonidine, and dexmedetomidine were used with local anesthetics due to their synergistic effects to improve the time to first rescue analgesia and sensory and motor block characteristics, to delay the onset of postoperative pain, and to reduce the requirement of local anesthetics. The use of narcotics is limited due to adverse effects such as respiratory depression, nausea, vomiting, and pruritus. Sedation, stable intra- and post-operative hemodynamics condition, minimum intraoperative blood loss, and prolonged postoperative analgesia are the main desirable qualities of an adjuvant in local anesthesia produced by clonidine and dexmedetomidine (1). Most studies in this regard agree that peripheral and central (spinal, epidural, and supraspinal) administration of clonidine or dexmedetomidine with local anesthetics provides good analgesia, sedation, sympatholysis, hypnosis, and anxiolysis (2).

Dexmedetomidine, the trade name Precedex, is a potent α2 adrenergic agonist with anxiety-reducing, sedative, and pain medication actions. The highly lipophilic nature of dexmedetomidine allows rapid absorption into the cerebrospinal fluid and binding to α2-AR and provides sympatholysis effect and anxiolytic effect and natural sleep effect with minimal respiratory depression. Clonidine is similar to dexmedetomidine, a potent adrenergic agonist a2 that is widely used to induce analgesia and sedation. Clonidine and dexmedetomidine are frequently used adjuvant to potentiate epidural and peripheral nerve block induced by local anesthetics and to improve the quality and duration of anesthesia. Although clonidine and dexmedetomidine are known as alpha-2 agonists and improve the quality and duration of blockade induced by local anesthetics, no study has been reported to compare their associated adverse events in local anesthesia. Therefore, the aim of this study was to make a comparison between the adverse events associated with the adjuvant use of local and intravenous dexmedetomidine and clonidine in local anesthesia. This comparison helps us in selection of alpha-2 agonists in adjuvant use for local anesthesia.

## Methods

### Database Search Strategy

A comprehensive search was performed to retrieve any reported adverse event associated with the use of dexmedetomidine and clonidine in local anesthesia from published literature. The following databases were searched since inception up to July 1, 2020; Medline, PubMed Central, EMBASE, Scopus, Cochrane Central Register of Controlled Trials, and Web of Science. Keywords including “dexmedetomidine,” “clonidine,” “trial,” “local,” “regional,” and “anesthesia” were used for database search. Our search was restricted to English language reports.

### Selection Criteria

To meet the study objective, human studies (including clinical trials, cohort studies, case-control studies, case reports, and series) on the use of dexmedetomidine and clonidine in local anesthesia from published literature were included. To be included in the meta-analysis studies should have reported the status of adverse events in two study groups (Table 1). The secondary reports, non-human studies, studies with no report on the adverse events were excluded.

**Table 1:** Characteristics of included studies in the meta-analysis.

**Trial year/country**	***n***	***n'***	**Procedure**	**Drug and route of administration**	**Jadad score**
Mukherjee et al. (3)	44	44	Breast cancer surgery	Adjuvant to ropivacaine in thoracic paravertebral block	5
Solanki et al. (4)	30	30	Trauma patients undergoing lower limb surgery	Adjuvant to intrathecal administration of bupivacaine	4
Panneer et al. (5)	30	30	Lower limb orthopedic surgeries	Adjuvant intravenous Dexmedetomidine and clonidine	5
Channabasappa et al. (6)	75	75	Modified radical mastectomy	Adjuvant to ropivacaine in cervical epidural anesthesia	5
Chiruvella et al. (7)	40	40	Total abdominal hysterectomies	Adjuvant to epidural levobupivacaine	4
Ganesh and Krishnamurthy (8)	50	50	Lower abdominal surgeries	adjuvant to intrathecal Bupivacaine	4
Kaur et al. (9)	60	60	Lower abdominal surgeries	Adjuvant intravenous dexmedetomidine and clonidine	4
Bajwa et al. (1)	25	25	Vaginal hysterectomies	adjuvant to epidural ropivacaine	5
Shaikh and Mahesh (2)	30	30	Lower limb orthopedic surgeries	Adjuvant to epidural bupivacaine	4
Agrawal et al. (10)	40	40	Fractures of lower limb	Adjuvant intravenous dexmedetomidine and clonidine	5
Li et al. (11)	21	21	Cesarean section	Adjuvant to intrathecal bupivacaine	5
Javahertalab et al. (12)	40	40	Lower limb orthopedic surgery	Adjuvant intravenous dexmedetomidine and clonidine spinal anesthesia with ropivacaine	4
Reddy et al. (13)	25	25	Orthopedic lower limb surgery	Adjuvant intravenous Dexmedetomidine and clonidine Spinal anesthesia bupivacaine	5
Sarma et al. (14)	50	50	Lower limb surgeries	Adjuvant to intrathecal bupivacaine	4

*n: number in the clonidine group*.

*n': number in dexmedetomidine group*.

### Data Extraction

Bibliographic information of all citations retrieved by the literature search was imported into Endnotes V.X6. Data extraction was done by two independent reviewers. Inconsistencies were resolved by a third reviewer.

Data were recorded on data extraction forms. Qualitative and quantitative data for the patient population, enrolment numbers in each group, reported adverse events in each group were extracted from each included study. The data were pooled for incidence rates for any adverse event during the study period regardless of causality association.

### Study Quality and Risk of Bias

Assessment of the quality of included studies was performed by the Jadad score.

### Statistical Analysis

A comparison of any reported adverse event was made between interventions by pooling data from studies using a direct meta-analysis technique. Heterogeneity between studies was assessed by Cochran's test (with a significance level of <0.1) and its composition using *I*^2^ statistics. If the *I*^2^ was higher than 50%, it indicates high heterogeneity. In the case of heterogeneity, the random-effects model was used with the inverse variance method. Dichotomous outcomes were summarized as odds ratios (OR). Subgroup analysis between intravenous administration and local administration was used to better indicate heterogeneity between studies. Comprehensive Meta-analysis version 2.0 software was used for the meta-analysis of the available data.

## Results

### Description of Search

**Figure 1:**
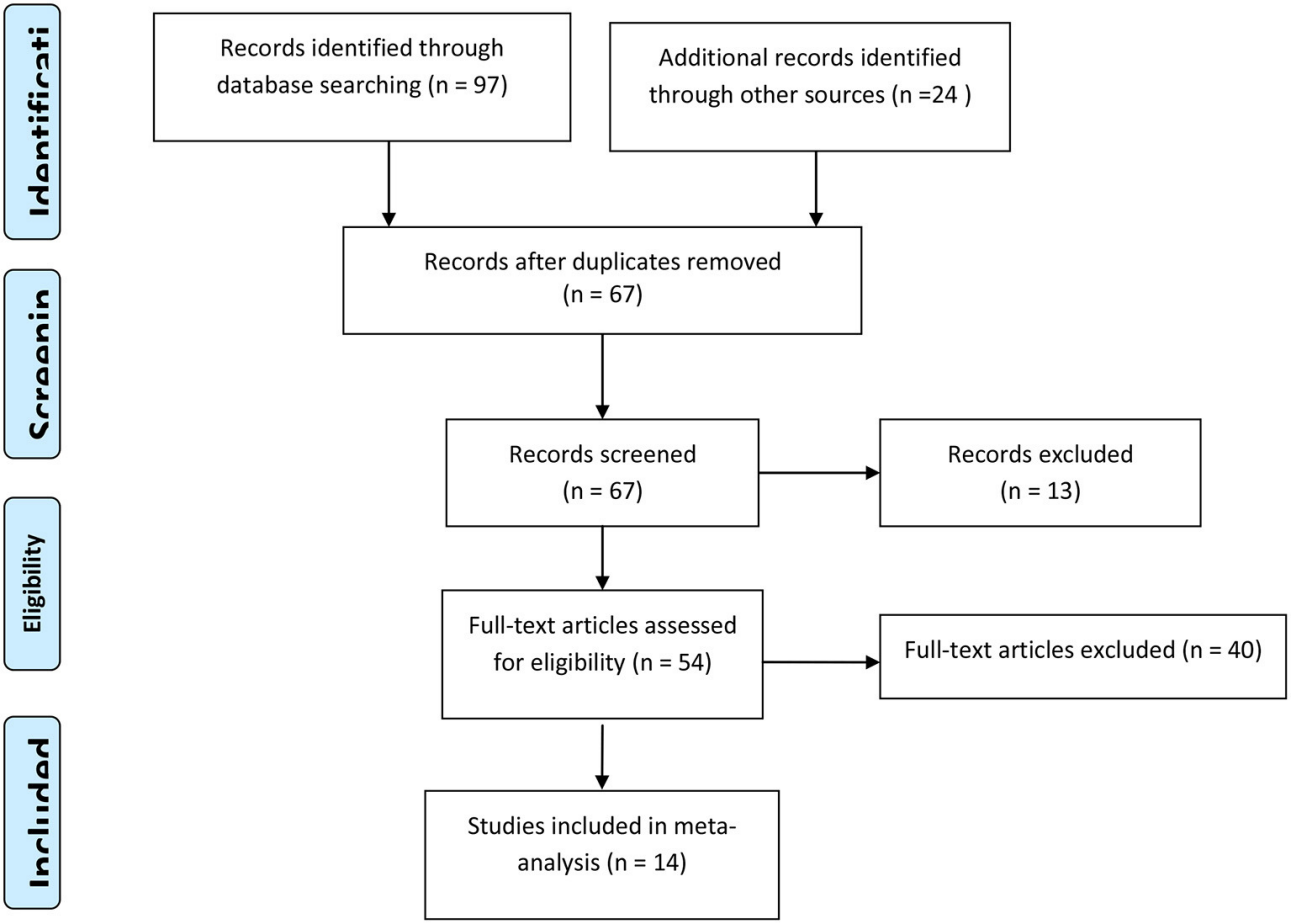
PRISMA flow diagram for included studies.

Searching the mentioned databases, from 121 retrieved articles finally 14 articles including 1,120 patients fulfilled the eligibility criteria to be included in the final analysis. The flowchart for study evaluation, selection, and inclusion is demonstrated in Figure 1.

### Characteristics of the Included Studies

The characteristics of the studies included in the meta-analysis are presented in Table 1. The details of the number of adverse events in each group are provided in Supplementary Table 1.

### Meta-Analysis and Data Synthesis Bradycardia/Hypotension

**Figure 2:**
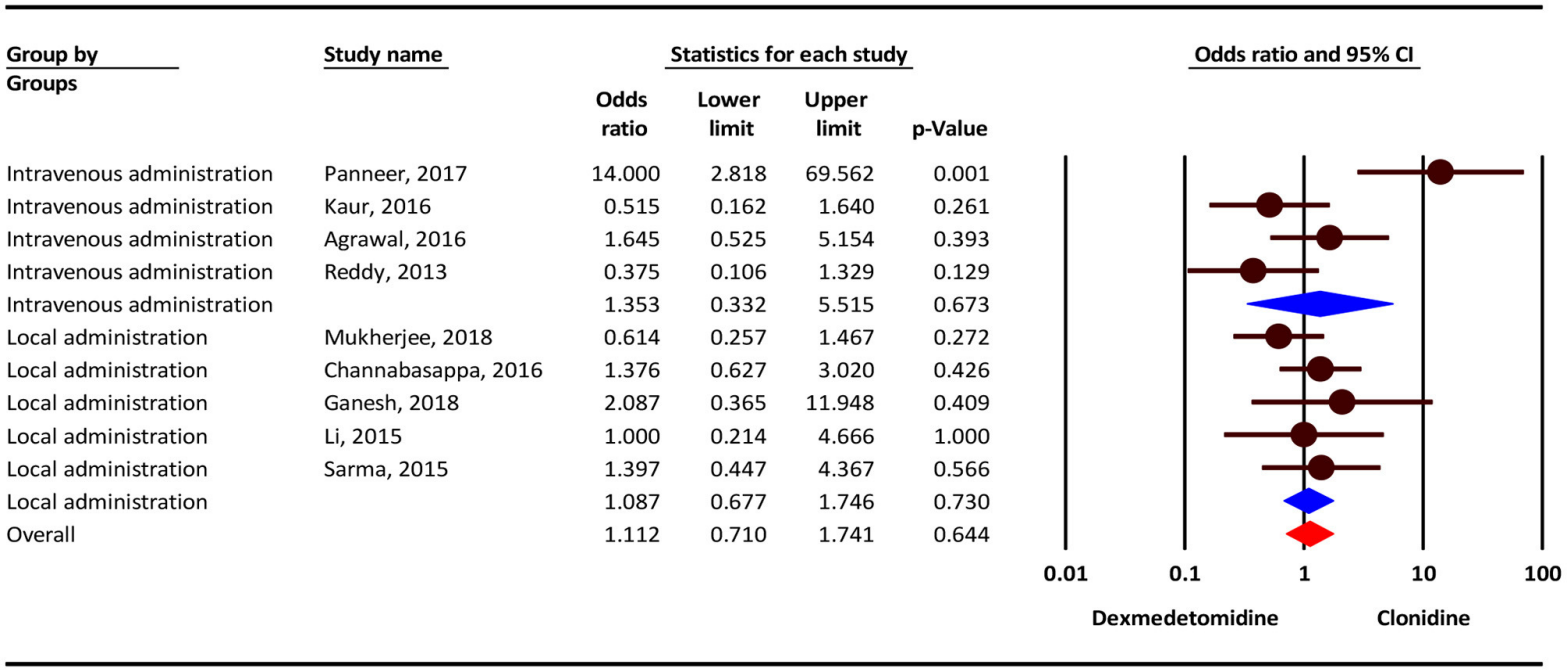
The odds ratio for the risk of Bradycardia/Hypotension as an adverse event associated with the adjuvant use of dexmedetomidine and clonidine in intravenous and local anesthesia.

According to the results, the severity of Bradycardia/Hypotension complication was 11% higher in the clonidine group, but this difference was not statistically significant (OR = 1.11; 95 % CI = 0.71–1.74; *P* = 0.644; *I*^2^ = 53.78 %, *P* = 0.027). Subgroup analysis show that, Bradycardia/Hypotension complicationin intravenous administration and local administration was 35 % (OR = 1.35; 95 % CI = 0.33–5.51, *P* = 0.673) and 8% (OR = 1.08; 95% CI = 0.67–1.74, *P* = 0.730) higher in the clonidine group, but this difference was not statistically significant (Figure 2).

### Nausea/Vomiting

**Figure 3:**
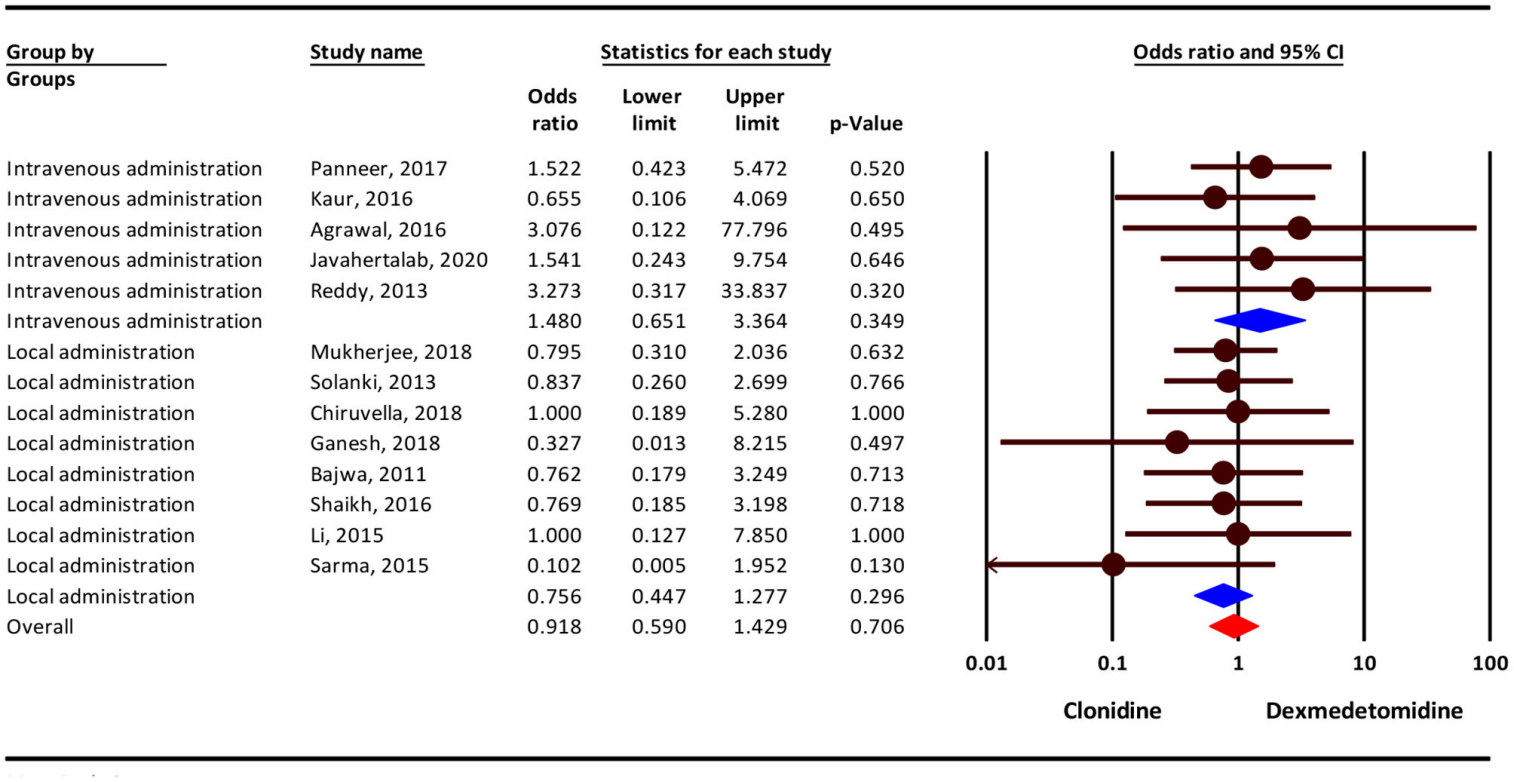
Odds ratio for the risk of Nausea/Vomiting as an adverse event associated with the adjuvant use of dexmedetomidine and clonidine in intravenous and local anesthesia.

Our result show that, the severity of nausea/vomiting complication was 9% lower in the clonidine group, but this difference was not statistically significant (OR = 0.91; 95 % CI = 0.59–1.42; *P* = 0.706; *I*^2^= 0.0%, *P* = 0.940). Subgroup analysis show that, nausea/vomiting complication in intravenousadministration was 48% higher in the dexmedetomidine group (OR = 1.48; 95% CI = 0.65–3.36, *P* = 0.349) and 25 % lower in the clonidine group (OR = 0.75; 95% CI = 0.44–1.27, *P* = 0.296), but this difference was not statistically significant (Figure 3).

### Dizziness/Headache

**Figure 4:**
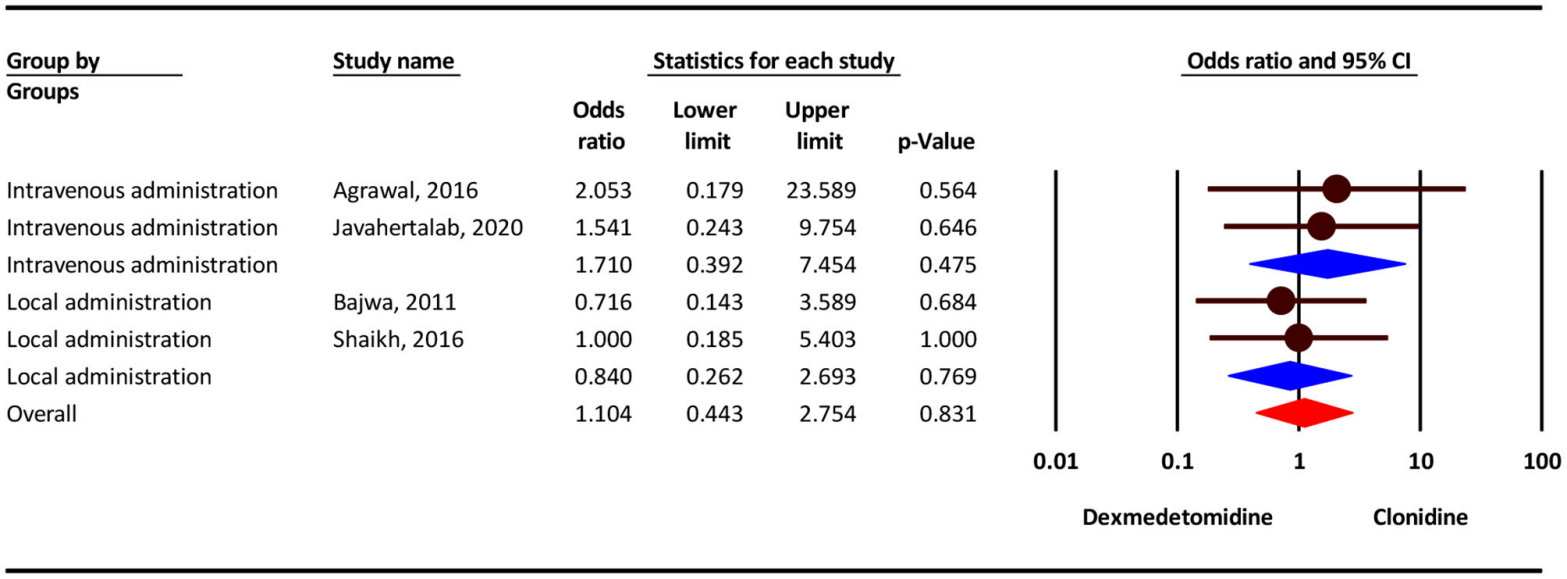
Odds ratio for the risk of Dizziness/Headache as an adverse event associated with the adjuvant use of dexmedetomidine and clonidine in intravenous and local anesthesia.

Our analysis show that, the severity of dizziness/headache complication was 10% higher in the clonidine group, but this difference was not statistically significant (OR = 1.10; 95% CI = 0.44–2.75; *P* = 0.831; *I*^2^ = 0.0%, *P* = 0.882). Subgroup analysis show that, dizziness/headache complication in intravenous administration and local administration was 71% higher in the clonidine group (OR = 1.71; 95% CI = 0.39–7.45, *P* = 0.475) and 16 % lower in the dexmedetomidine group (OR = 0.75; 95% CI = 0.26–2.69, *P* = 0.769), but this difference was not statistically significant (Figure 4).

### Shivering

**Figure 5:**
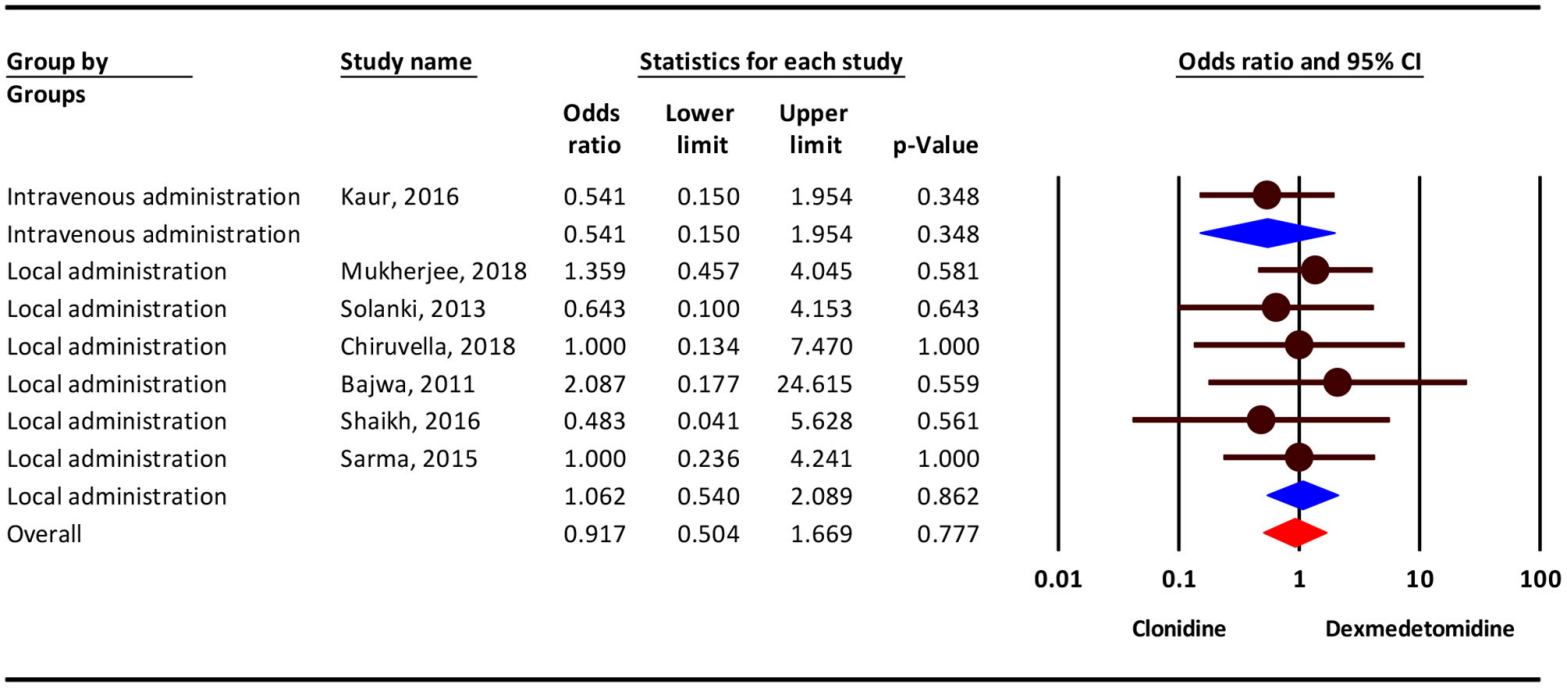
Odds ratio for the risk of shivering as an adverse event associated with the adjuvant use of dexmedetomidine and clonidine in local anesthesia.

Based on our results, the severity of shivering complication was 9% lower in the clonidine group, but this difference was not statistically significant (OR = 0.91; 95% CI = 0.50–1.66; *P* = 0.831; *I*^2^ = 0.0%, *P* = 0.920). Subgroup analysis show that, dizziness/headache complication in intravenous administration was 46 % lower in the clonidine group (OR = 0.54; 95% CI = 0.15–1.95, *P* = 0.348) and in local administration 6% higher in the dexmedetomidine group(OR = 1.06; 95% CI = 0.54–2.08, *P* = 0.862), but this difference was not statistically significant (Figure 5).

### Dry Mouth

**Figure 6:**
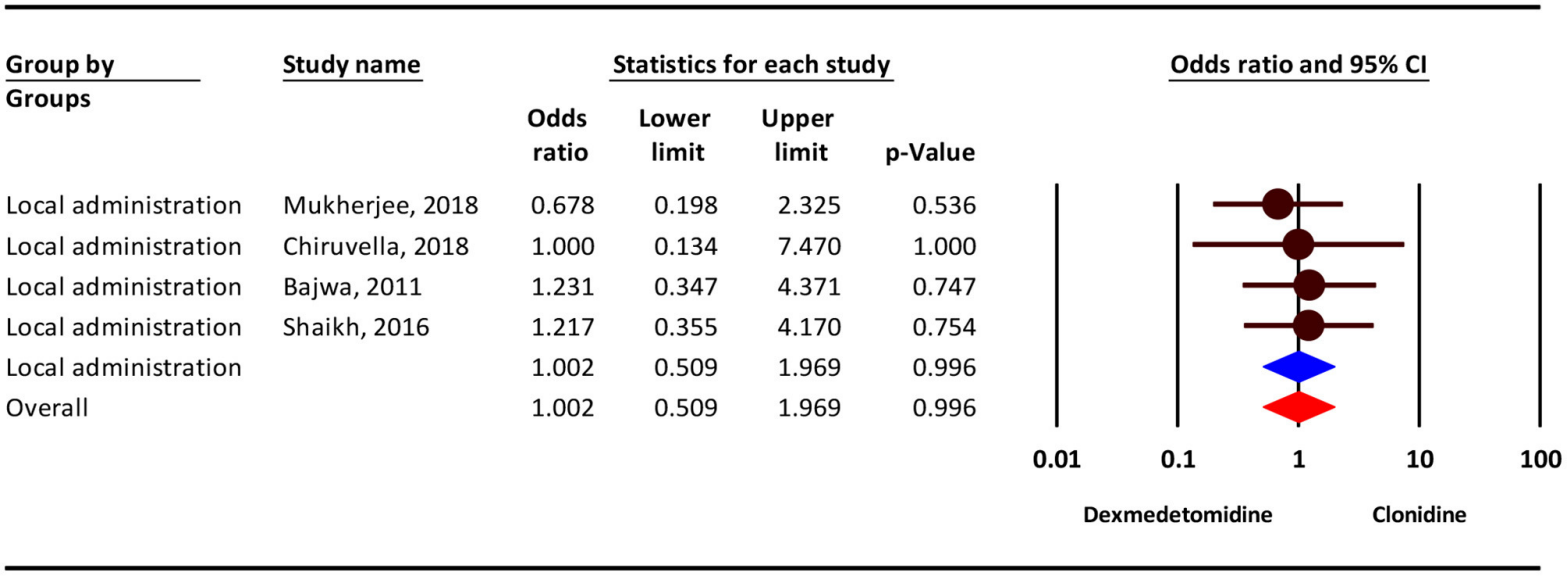
The odds ratio for the risk of dry mouth as an adverse event associated with the adjuvant use of dexmedetomidine and clonidine in local anesthesia.

Our analysis show that, the severity of dry mouthcomplication showed no difference between the two groups in local administration (OR = 1.00; 95% CI = 0.50–1.96; *P* = 0.996; *I*^2^ = 0.0%, *P* = 0.900) (Figure 6).

### Publication Bias

**Figure 7:**
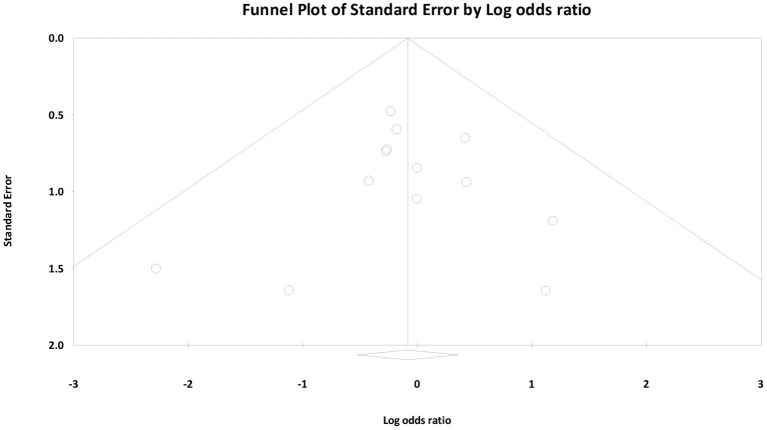
Funnel plots to explore the possibility of publication bias.

Finally, we created funnel plots to explore the possibility of publication bias, yet the results of Egger's test were not evidence of this bias (bias: −0.62, 95% CI = −1.30 to 1.18; *P* = 0.914) (Figure 7).

## Discussion

In this systematic review and meta-analysis study, we analyzed the findings from 14 randomized trials to compare the adverse events associated with the adjuvant use of clonidine and dexmedetomidine in local anesthesia. The reported adverse events include cardiorespiratory events, dizziness, headache, nausea and vomiting, shivering, and dry mouth. In this study, data extracted from the included studies were analyzed by fixed and random effect models. The results of this analysis showed no significant difference in the rate of adverse events reported for clonidine and dexmedetomidine in local and intravenous use in anesthesia.

Evidence shows that adjuvant use of alpha-2 adrenoreceptor agonists improves the performance of local anesthetics through induction of longer postoperative analgesia and minimizing intraoperative blood loss and postoperative side effects. Adjuvants such as clonidine and dexmedetomidine were used with local anesthetics due to their synergistic effects to improve the time to first rescue analgesia and sensory and motor block characteristics, to delay the onset of post-operative pain, and to reduce the requirement of local anesthetics. Sedation, stable intra- and post-operative hemodynamics condition, minimum intraoperative blood loss, and longer duration postoperative pain control are the main desirable qualities of an adjuvant in local anesthesia produced by clonidine and dexmedetomidine (1). Most studies in this regard agree that peripheral and central (spinal, epidural, and supraspinal) administration of clonidine or dexmedetomidine with local anesthetics provides good analgesia, sedation, sympatholysis, hypnosis, and anxiolysis (2).

Clonidine as a partial α2-adrenergic agonist potentiates both sensory and motor block of local anesthetics. An elimination half-life of clonidine is 14.6 h and dexmedetomidine is 2 h. The half-life of dexmedetomidine ranged from 2.1–3.7 h while clonidine has a wider range of 6–23 h (15). Therefore, plasma concentrations of these drugs are affected by the normal function of the liver and kidneys.

Physiologically, some of the adverse events can be explained by the central role of these drugs. Clonidine, for example, has the ability to cross the blood-brain barrier and can be readily available to the brain nuclei and centers such as the prefrontal cortex or especially the vasomotor center in the brainstem that controls nausea, blood pressure, and heart rate (16, 17).

The differences in the results of some studies may be due to the use of different pretreatment drugs, differences in the study method, type of surgical procedures, and pharmacological properties of these two drugs. Moreover, it should also be noted that many of the side effects observed in these studies are not only due to the local anesthetic used but also to the type of surgery.

The results of this study showed that the use of clonidine and dexmedetomidine as an adjuvant to local anesthetics had no significant effects on adverse events such as bradycardia, dizziness, nausea, dry mouth, and shivering in patients under local anesthesia. Most studies in this regard agree that the use of clonidine or dexmedetomidine with local anesthetics has no significant effect on cardiorespiratory parameters such as HR and MAP and other side effects such as headache and respiratory depression in patients under local anesthesia (1, 6, 8–10, 13). However, some studies have found conflicting findings (12).

Solanki et al. reported that the addition of dexmedetomidine or clonidine to intrathecal bupivacaine had no significant effects on nausea, bradycardia, and hypotension either during or after surgery (4). In a study, Channabasappa et al. showed that the mean values of hypotension, bradycardia and respiratory depression were not significantly different between the two groups of dexmedetomidine and clonidine throughout the intraoperative and postoperative periods (6). Chiruvella et al. showed that the addition of intravenous dexmedetomidine or clonidine to levobupivacaine had no significant effects on the incidence of hypotension, bradycardia, dizziness, respiratory depression, nausea, vomiting, and shivering in patients during or after surgery (7). In contrast, Ganesh and Krishnamurthy reported that the addition of dexmedetomidine and clonidine to bupivacaine cause a significant difference in HR between dexmedetomidine and clonidine groups at 5, 10, and 15 min of follow up (8). These effects may be due to the effects of acute intra or post-operative pain on the outflow of the sympathetic system, where clonidine is weaker than dexmedetomidine. They also reported that there was a significant difference in mean systolic blood pressure between dexmedetomidine and clonidine groups at 50 min of follow-ups (8).

Studies showed that cardiovascular depression and gastrointestinal side effects such as nausea are the common complications of the subarachnoid block in patients under local anesthesia or general anesthesia (10). These changes can be explained by the pharmacodynamic properties of dexmedetomidine and clonidine on the brain, especially the center of vasomotor control, and suppressive effect on the central sympathetic drive (16, 18) which can also decrease circulating levels of norepinephrine. Some researchers believe that the use of clonidine and dexmedetomidine induces more cardio-respiratory depression (10, 19). They suggested a lower incidence of nausea, vomiting in clonidine and lower sedative effect in dexmedetomidine receivers. Although the results of our study did not show a significant difference in the evaluation of these parameters in dexmedetomidine and clonidine, a definite conclusion in this regard requires further studies. Overall, the findings of the previous studies (2, 20, 21) demonstrated that the cardiorespiratory parameters remain stable intra- and postoperative periods with slight changes, which confirms the beneficial effects of α-2 agonists in providing a stable period. Our results showed no significant difference in cardiovascular depression and gastrointestinal side effects between the adjuvant use of dexmedetomidine and clonidine.

There is a high incidence rate of shivering following both general and regional anesthesia (22). Different causes are explained with peri-operative hypothermia as the most important cause. Shivering is associated with peri-operative sympathetic nervous system stimulation, delayed recovery, metabolic acidosis, bleeding disorders, and immune system dysfunction (22). Our results showed no significant difference in postoperative shivering between the adjuvant use of dexmedetomidine and clonidine.

Despite the clear findings of this meta-analysis, our study had important limitations and confounding factors. The most important limitation was the lack of a uniform definition of some of the assessed adverse events.

To sum up, our cumulative analysis of all available data in the field showed that there is no significant difference in adverse events associated with the adjuvant intravenous or local use of clonidine and dexmedetomidine in local anesthesia. The reported adverse events included bradycardia, hypotension, respiratory depression, dizziness, headache, nausea and vomiting, shivering, and dry mouth. Future research should be designed to better evaluate the outcomes of adjuvant use of alpha-2 agonists with more controlled condition and larger sample size. The mechanism associated with the observed adverse effects should be also more investigated in future studies.

## Data Availability Statement

The original contributions presented in the study are included in the article/Supplementary Material, further inquiries can be directed to the corresponding author/s.

## Author Contributions

HS and XM made the first inception of the study and developed the proposal. JJ, JZ, ZW, and XS searched the databases and extracted the data. JJ, JC, and BZ analyzed the data. HS wrote the first draft of the manuscript.

## Conflict of Interest

The authors declare that the research was conducted in the absence of any commercial or financial relationships that could be construed as a potential conflict of interest.
