# Supplementary material for: Comparative Study of the Adverse Events Associated With Adjuvant Use of Dexmedetomidine and Clonidine in Local Anesthesia
Source: Front Med (Lausanne). 2021 Jun 24;8:602966. doi: 10.3389/fmed.2021.602966 (PMC8264190; doi:10.3389/fmed.2021.602966)
Supplement: Supplementary file 1 [file Table_1.docx]

Supplementary table 1. Number of adverse events in each group in the included studies

| Study | n | n’ | Dizziness/Headache | | Nausea/Vomiting | | Bradycardia/Hypotension | | Shivering | | Dry mouth | | Respiratory depression | | Pruritus | | Sedation | |
| --- | --- | --- | --- | --- | --- | --- | --- | --- | --- | --- | --- | --- | --- | --- | --- | --- | --- | --- |
|  | C | D | C | D | C | D | C | D | C | D | C | D | C | D | C | D | C | D |
| Mukherjee et al., 2018 | 44 | 44 |  |  | 11 | 13 | 25 | 30 | 9 | 7 | 5 | 7 |  |  |  |  | 10 | 15 |
| Solanki et al., 2013) | 30 | 30 |  |  | 7 | 8 |  |  | 2 | 3 |  |  |  |  |  |  |  |  |
| Panneer et al., 2017) | 30 | 30 |  |  | 7 | 5 | 15 | 2 |  |  |  |  | 0 | 0 |  |  |  |  |
| Channabasappa et al., 2016 | 75 | 75 |  |  |  |  | 18 | 14 |  |  |  |  | 2 | 0 |  |  |  |  |
| Chiruvella et al., 2018) | 40 | 40 | 0 | 0 | 3 | 3 | 0 | 0 | 2 | 2 | 2 | 2 | 0 | 0 |  |  |  |  |
| Ganesh and Krishnamurthy, 2018) | 50 | 50 |  |  | 0 | 1 | 4 | 2 | 0 | 0 |  |  |  |  |  |  |  |  |
| Kaur et al., 2016) | 60 | 60 |  |  | 2 | 3 | 5 | 9 | 4 | 7 |  |  | 0 | 0 |  |  |  |  |
| Bajwa et al., 2011) | 25 | 25 | 3 | 4 | 4 | 5 |  |  | 2 | 1 | 7 | 6 | 0 | 0 |  |  |  |  |
| Shaikh and Mahesh, 2016 | 30 | 30 | 3 | 3 | 4 | 5 |  |  | 1 | 2 | 7 | 6 | 0 | 0 |  |  |  |  |
| Agrawal et al., 2016 | 40 | 40 | 2 | 1 | 1 | 0 | 9 | 6 |  |  |  |  | 2 | 5 | 0 | 0 |  |  |
| Li et al., 2015 | 21 | 21 |  |  | 2 | 2 | 4 | 4 |  |  |  |  | 0 | 0 | 1 | 1 |  |  |
| Javahertalab et al., 2020) | 40 | 40 | 3 | 2 | 3 | 2 | 0 | 0 |  |  |  |  |  |  |  |  |  |  |
| Reddy et al., 2013) | 25 | 25 |  |  | 3 | 1 | 5 | 10 |  |  |  |  |  |  |  |  | 6 | 17 |
| Sarma et al., 2015 | 50 | 50 |  |  | 0 | 4 | 8 | 6 | 4 | 4 |  |  | 0 | 0 | 0 | 0 |  |  |

n: number in the clonidine group

n’: number in dexmedetomidine group

C: clonidine

D: dexmedetomidine
