# Supplementary material for: Comparative Study of the Adverse Events Associated With Adjuvant Use of Dexmedetomidine and Clonidine in Local Anesthesia
Source: Front Med (Lausanne). 2021 Jun 24;8:602966. doi: 10.3389/fmed.2021.602966 (PMC8264190; doi:10.3389/fmed.2021.602966)
Supplement: Supplementary file 2 [file Data_Sheet_1.docx]

Search strategy:

(dexmedetomidine[Title] AND clonidine[Title]) AND ("trial"[Title/Abstract] OR "local"[Title/Abstract] OR "regional"[Title/Abstract] OR "anesthesia"[Title/Abstract])
